# Health and social care: The link between social care deficiencies and health care pressures

**DOI:** 10.12968/bjhc.2023.0080

**Published:** 2024-05-06

**Authors:** S Mechie, K Ujhelyi Gomez, J Harrison, JE Hill

**Affiliations:** Lancashire County Council; University of Liverpool; University of Central Lancashire

**Keywords:** social support, delivery of health care, economics, systematic review, health equity

## Abstract

In the United Kingdom, demand on the social care sector is rising coupled with a significant reduction in funding making it difficult to meet these needs. The aim of this two-part commentary is to provide a critical evaluation of the evidence base in relation to solutions to tackle the growing demands on health and social care. Part 1 focuses on the evidence from a systematic review around the association between the availability and supply of social care and healthcare on utilisation for older adults in high income countries.

**Part 1. Health and social care: The link between social care deficiencies and health care pressures:**

An evidence summary based on the following systematic review:

Spiers G, Matthews FE, Moffatt S, Barker RO, Jarvis H, Stow D, Kingston A, Hanratty B (2019) Impact of social care supply on healthcare utilisation by older adults: a systematic review and meta-analysis. *Age and Ageing* 48(1):57-66. https://doi.org/10.1093/ageing/afy147

## Introduction

Social care in the United Kingdom is a large and varied sector which supports individuals with a disability or physical/mental illness both at home and in care settings to facilitate independent functioning and to improve wellbeing (DoH, 2017). It is a sector experiencing increasing demands as today’s population lives longer and has complex and often long-term health issues (Kingston et al, 2018). Since 1981, there has been a 52% rise in the number of people aged 65 and over and this number is expected to further increase by almost a third in the next two decades (Centre for Aging Better, 2022).

With age, health deteriorates and the need for health and social care support rises with an expected further increase in the future (Ferguson and Belloni, 2019). However, while the demand is rising, social care has experienced a significant reduction in funding making it difficult to meet these needs (Care Quality Commission, 2016). Moreover, such needs are even higher in more deprived areas with higher funding cuts (Liverpool City Council, 2017). Estimates show that about 1.5 million people aged 50 and over in England are not receiving adequate social care (Age UK, 2019). At the same time, there is evidence to show that insufficient supply of social care service places pressure on the health sector (NAO, 2017), such as delayed discharge from hospitals and increased readmissions (Limb, 2022).

To support the need for funding and an increasing policy focus in these areas, evidence of a direct association between social care resources and health service demands was needed. To fill this gap, Spiers et al (2019) completed a systematic review with the aim to identify evidence for the relationship between the availability and supply of social care and healthcare on utilisation for older adults in high income countries. Another review by Baxter and colleagues (2018) looked at the international literature around integrated care models and their outcomes to help design a health and social care system in England that is both financially sustainable and delivered in line with patients’ health needs and personal preferences.

### Aims of commentary

This commentary has been separated into two parts. This two-part series commentary aims to critically appraise the reviews by Spiers et al (2019) (part one) and Baxter et al (2018) (part two) and contextualise the findings to practice in relation to solutions to tackle the growing demands on health and social care in the UK.

### Methods of Spiers et al. (2019)

This protocol registered systematic review conducted an extensive literature search using multiple databases from inception of each database to October 2016 including a final update in May 2018. Additionally, hand searches of references from included studies were undertaken. Although time restrictions were not applied to the search, studies conducted prior to 2000 were excluded due to concerns around relevance.

All studies met the inclusion criteria if they were of experimental, quasi-experimental or observational design, conducted in high-income countries and examined the link between an indicator of social care accessibility and healthcare utilisation. Social care accessibility was defined as social care supply and availability such as a measure of social care provision relative to the potential client population, (e.g. number of care home beds per 1000 adults). Healthcare utilisation referred to contact with and use of all primary and secondary healthcare services. Participants were older adults over 60.

Abstract titles and full papers were screened by one author and a sub-sample (50%) was checked by two other researchers. An online software tool (Rayyan) was also used to both identify potentially relevant publications and manage the screening process. Full-text papers were assessed and study data extracted from each included paper. Assessment of bias was undertaken using the National Institute of Health Quality Assessment Tool for Observational Cohort and Cross-Sectional Studies.

The authors stated that due to the small number of studies associated with each outcome, a formal meta-analytical approach was not possible. To provide a graphical representation of the findings, a random-effects meta-analysis summary estimate was calculated.

### Findings of Spiers et al. (2019)

The review included 12 studies all of which used a cross sectional design. Studies were from the UK (7), Italy (2), USA (2) and Norway (1). The authors rated seven of the included studies as good quality, one as fair and four as poor. Studies rated as good quality, had adjusted their analysis for potential confounders. Three of the four studies rated as poor were included while one was excluded from the analysis as it was not possible to extract data.

Results from the 12 studies reporting on cross-sectional secondary analyses of administrative data around the evidence for the relationship between social care availability and supply and health care utilisation were reported in four sections: delayed discharge; length of hospital stay; admissions/re-admission and emergency service use; and healthcare expenditure.

#### Delayed discharge

Three studies found statistically significant association between a higher number of available care home beds and reduction in delayed discharge and the number of people experiencing a delay. Two of these studies were rated as ‘good’ quality. One study was rated as ‘poor’ due to data limitations. Two studies found that a reduction in delayed discharge was statistically significantly associated with higher social care expenditure. While both of these studies were rated as good quality, limitation to the data was noted in one of the studies.

#### Length of hospital stay

A statistically significant relationship was found between shorter length of hospital stay for the older population and increased availability of long-term care home beds in three studies with mixed quality (‘good’, ‘fair’, and ‘poor’). One study with ‘fair’ and one with ‘poor’ rating had inconsistent findings regarding the effect of homecare on length of stay.

#### Admissions, readmissions and emergency service use

A statistically significant relationship was demonstrated by three studies (two ‘good’ quality, one ‘poor’) between reduction in emergency readmissions and greater availability of care homes, but findings regarding the effects of social care expenditure on readmissions or emergency admissions were inconsistent or limited. There was little evidence related to the influence of homecare hours on emergency readmissions.

#### Healthcare expenditure

increased care home expenditure was statistically significantly linked to reduction in hospital spending in two studies, one rated as ‘good’ quality and one as ‘poor’.

No sub-group or sensitivity analyses were conducted.

### Commentary

According to the Joanna Briggs Institute Critical Appraisal tool for systematic reviews (Aromataris et al, 2015), only four out of the 11 criteria were judged to be satisfactory for this review. The review question was not explicitly stated and only a very broad aim was provided. Although the quality appraisal tool used was appropriate for the appraisal of observational cohort and cross-sectional studies, a more relevant tool may have been more appropriate to assess the two cost-effectiveness studies. It was unclear who performed the critical appraisal of included studies. Four studies were rated as poor quality, of which three were included, which may have impacted on the quality of the findings. It was not specified who performed the data extraction, and therefore, errors at this step could have occurred. Furthermore, two studies lacked sufficient data but only one of them was excluded from the analysis. Data synthesis methods were not clearly reported. Finally, publication bias was not considered or assessed.

These limitations and the exploratory observational nature of the primary data mean that the findings of this review are subject to substantial bias. Due to the quality issues of the review, the scarcity of evidence around the relationship between social care supply and healthcare utilisation, and the high heterogeneity of variables, it was deemed that the accuracy of this systematic review and the applicability of these findings are limited.

The review findings suggest a reduction in healthcare expenditure as a result of an increase in social care expenditure, but this finding is limited as no formal economic evaluation was performed. There is some evidence for the benefits of care home support over discharge to the home, although home-care related research is scarce. Guidelines recommend the ‘*discharge to assess, home first*’ approach (DoH, 2022), where the majority of patients are sent home upon discharge (rather than to a care home). Therefore, further research using a robust randomised and controlled design is essential to investigate this area.

Within the review, ten studies adjusted for variables important to identify health inequalities in accessing social care, such as personal characteristics, area deprivation, and wealth. However, as noted by the authors of the review, only a few studies analysed population sub-groups, providing little evidence for group differences in healthcare use affected by the availability and supply of social care. Further investigation is needed to determine how some populations may be affected differently, such as people living in deprived areas where demand is higher due to poorer health, or people who need an interpreter to communicate their needs, both leading to longer waiting times. The review also highlighted a need for research examining the link between social support supply and primary care which was suggested to be an important sector to identify unmet social care needs.

Good quality primary research investigating the effectiveness of complex interventions used in social care is rare. Therefore, reviews synthesising evidence arising from such studies do not normally meet the standards of systematic reviews and it may be more difficult to draw reliable conclusions to inform practice. It has been suggested that traditional systematic review methods may not be the best choice to synthesise such evidence (Pawson et al, 2005). Additionally, a project advisory group involving a range of different stakeholders might be better placed than researchers alone to formulate relevant recommendations to practice (Rutter, 2013).

While the increase of social care supply may ease the pressures on the healthcare system through reducing the delay in discharge from hospital and the percentage of readmissions, the findings of this review do not provide quantifiable evidence to support this. Nevertheless, it warrants further investigation of the association between availability of social care supply and healthcare utilisation through better quality primary studies that use comparison groups and can provide stronger data for future systematic reviews.

## Key points

1. Increase of social care supply in the United Kingdom may reduce the pressures on the health care system. However, quantifiable evidence to support this is missing
2. Research evidence is lacking around primary care healthcare use, differences of impact between population sub-groups, and home-based care.
3. Further investigation of the association between availability of social care supply and healthcare utilisation is warranted through better quality primary studies that use comparison groups and can provide stronger data for future systematic reviews.
4. Further investigation is needed to determine how some populations may be affected differently, such as people living in deprived areas.

## Questions for thoughts

1) Why is it difficult to synthesise social care-related evidence?
2) How could we improve the synthesis of research evidence in social care?
3) What kind of research evidence would better inform your practice?
